# Digitalization and Automation of Runway Inspection Using Unmanned Aerial Vehicles

**DOI:** 10.3390/s26041100

**Published:** 2026-02-08

**Authors:** Marios Krestenitis, Alexandros Petropoulos, Ilias Koulalis, Irina Stipanovic, Sandra Skaric Palic, Konstantinos Ioannidis, Stefanos Vrochidis

**Affiliations:** 1 Centre for Research and Technology-Hellas (CERTH), 57001 Thessaloniki, Greece; 2 Infra Plan Consulting, 10000 Zagreb, Croatia; 3 Faculty of Engineering Technology, University of Twente, 7522 NB Enschede, The Netherlands

**Keywords:** airport runway inspection, AI-based defect detection, predictive maintenance, digital twins, UAVs

## Abstract

This paper presents an end-to-end framework for automated inspection and condition assessment of airport runway pavement using UAV-acquired imagery. The proposed approach integrates Unmanned Aerial Vehicle (UAV)-based data collection, deep learning-based pixel-level semantic segmentation of surface defects, and Geographic Information System (GIS)-based spatial aggregation to generate a georeferenced digital representation of airfield pavement condition. Multiple safety-critical defect types are detected and localized at pixel resolution, while spatially referenced processing enables a Pavement Condition Index (PCI)-inspired condition assessment based on defect density within predefined sampling units. The framework is validated through a real-world case study at Zadar Airport, where the entire runway was surveyed using high-resolution UAV imagery. The results demonstrate the system’s capability to identify and map multiple defect categories across the full runway extent and to produce a coherent, runway-scale condition map supporting maintenance prioritization and decision-making. Overall, the proposed solution provides a scalable, data-driven alternative to traditional manual runway inspection workflows and establishes a practical foundation for digital condition monitoring of airport pavement infrastructure.

## 1. Introduction

The digitalization of transport infrastructure, particularly through the development of Digital Twins (DTs), has gained increasing attention in recent years as a means of supporting operational and maintenance management practices. Digital twins offer significant opportunities to enhance lifecycle management by integrating geometric, material, and operational information with automated inspection workflows, enabling timely and evidence-based decision-making across assets such as bridges, pavements, and airport infrastructure [1,2,3]. In parallel, UAV-based imaging and deep-learning methods have matured into practical tools for large-area condition assessment, especially in scenarios where manual inspection is labor-intensive, hazardous or inconsistent and prone to subjectivity [4,5,6,7].

Within this context, airport runways represent a critical infrastructure asset where condition monitoring is critical to ensure operational safety and effective maintenance planning. Runway surface defects directly affect safety-critical factors such as aircraft braking performance. Timely detection and continuous monitoring of these defects, together with accurate evaluation of pavement condition, are required to support targeted maintenance activities. Recent work has demonstrated the feasibility of embedding inspection data and maintenance indicators within a digital-twin environment to support runway condition monitoring and decision-making [8,9]. However, for such platforms to function as effective maintenance decision-support tools, they must provide reliable, detailed, and automated condition assessments.

Despite the advances in UAV-based inspection and computer vision techniques, most existing approaches either focus on isolated defect classes or produce qualitative condition indicators that are difficult to reconcile with established engineering assessment procedures such as the Pavement Condition Index (PCI) [10,11]. Standardized under ASTM D5340, PCI remains the industry reference for airfield pavement condition assessment and maintenance prioritization [12], linking defect type, severity, and density to actionable maintenance decisions [13]. Although recent studies have demonstrated that PCI-oriented metrics can be derived from UAV imagery [6], accurate and operationally meaningful assessment requires precise pixel-level defect segmentation and robust defect density estimation.

To address this gap, this paper presents an end-to-end framework that integrates UAV-based data acquisition, AI-driven semantic segmentation, and GIS-based spatial analysis to support automated, PCI-inspired runway condition assessment. The proposed method processes UAV imagery to detect and localize multiple runway surface defect types at pixel resolution and aggregates the resulting georeferenced information within a GIS-based asset management environment. This integrated representation provides a quantitative support for maintenance planning and decision-making and serves as a practical digital twin component for airfield pavement condition assessment and maintenance prioritization.

## 2. Background

### 2.1. Runway Inspection

Visual inspection of runways is a crucial aspect of airport safety and maintenance management. It involves regular and systematic inspections of runway surfaces and associated components to ensure compliance with operational safety requirements during aircraft landing, take-off, taxiing, and ground handling. Such inspections are mandated by aviation regulations, including ADR.OPS.B015 of Regulation (EU) 139/2014, which requires continuous monitoring of airport operational areas to ensure safe aircraft operations [14].

The frequency of these inspections varies depending on their purpose, ranging from routine inspections conducted several times per day to more detailed condition surveys performed periodically. A primary focus of these inspections is the condition of the runway surface, including the identification of surface distress, foreign object debris (FOD), and other defects that may compromise operational safety. Routine visual inspections are typically carried out by trained airport personnel using inspection vehicles [15]. Inspectors are tasked with identifying potential hazards that could endanger the safe landing and take-off of aircraft. Any observed anomalies are documented using digital cameras, providing visual evidence that supports operational managers in maintenance documentation, planning, and decision-making.

Formal pavement condition assessment is commonly performed in accordance with standardized procedures such as ASTM D5340-20, which defines the Pavement Condition Index (PCI) methodology for evaluating pavement distress types, severity, and extent based on visual inspection [12]. These inspections require systematic observation and documentation of multiple distress categories, including cracking, surface deformation, and material degradation. In addition to structural distress, surface friction characteristics are a critical safety parameter for runways, as they directly influence aircraft braking performance during landing operations.

Tyre marks represent a significant factor affecting runway surface friction and, consequently, operational safety. Accumulation of rubber deposits can reduce skid resistance, particularly in touchdown zones, necessitating regular monitoring, friction testing, and cleaning operations to restore acceptable friction levels. Effective detection and monitoring of tyre marks are therefore essential for preventing safety incidents, maintaining compliance with regulatory friction requirements, and supporting proactive runway maintenance strategies [16,17,18,19,20,21].

Maintenance planning is a systematic process that involves the systematic identification and prioritization of observed pavement defects to ensure continued operational safety while minimizing service disruptions and lifecycle costs. While pavement deterioration due to operational use and environmental exposure is unavoidable, timely and data-driven maintenance interventions can significantly mitigate safety risks and extend pavement service life [16]. In this context, digital twin-oriented approaches that encompass historic information on damage recordings, damage observations, and maintenance activities provide a structured foundation for optimized runway maintenance planning and condition-based asset management.

### 2.2. Defect Detection

The detection of surface defects in visual data has attracted significant attention from the computer vision and machine learning research communities. Automated visual recognition of surface defects, typically cracks, presents a significant challenge due to their irregular shape and size, and their essential similarity to the background texture. Furthermore, case-to-case variations of the background add further limitations to deriving an “all-inclusive” solution that can be generalized in most infrastructures and construction materials.

Early approaches to automated crack detection primarily relied on classical image processing techniques aimed at enhancing crack-like structures and discriminating them from background textures. Tree structures [22] and genetic algorithms [23] have been utilized towards this direction. A substantial body of work focused on image filtering, aiming to facilitate the distinction of the crack instances from the background. The authors of [24] proposed a method utilizing Gabon filters to detect cracks on varying-texture pavement images. Authors of [25] proposed a method combining image filtering to reduce noise and enhance crack-related features with a probabilistic model to detect the cracks depicted in the processed image. In [26], an edge detection method was presented, aiming to identify crack-like edges through Hessian matrix-based filtering of the image.

With the advent of Convolutional Neural Networks (CNNs), learning-based approaches have increasingly dominated the field of surface defect detection. A deep learning architecture, named GoogleNet, was utilized in [27] to classify surface cracks in high-resolution images. Similarly, authors of [28] exploited a custom-built dataset to train a CNN model capable to detect cracks on pavement images. CrackNet was proposed in [29] as a CNN-based method capable of preserving the spatial dimensions of the input image to detect the depicted cracks at the pixel level. Based on SegNet [30], DeepCrack was introduced in [31], a deep learning architecture which semantically segments the input image into crack and background. Similar approaches, based on implementing semantic segmentation, were also employed in [32,33], aiming to detect crack instances in road and pavement surfaces.

A growing body of research has explored the use of UAV-acquired visual data for automated surface defect detection in civil infrastructure. The authors of [34] deployed a crack detection method for UAV-based bridge inspection. The approach relied on a sophisticated image processing framework to enhance the contrast between crack and background, leading to efficient detections. Learning-based methods have subsequently been introduced, including CNN-based frameworks for crack detection in UAV imagery for building inspection [35]. Authors of [36] developed a CNN-based framework that processes UAV images, classifying them into categories of ‘crack’ or ‘no crack’. By combining other sensing modalities, this framework provides information regarding the location of the detected cracks. More recently, interactive inspection platforms have been proposed that integrate UAV-based image acquisition with high-resolution 3D scene representations and computer vision-based defect detection, enabling richer visual exploration of inspected assets [37].

In parallel, recent studies have investigated the use of Visual–Language Models (VLMs) for pavement condition assessment by associating visual inputs with textual descriptions of surface damage. Krestenitis et al. [7] introduced this idea by proposing a CLIP-based [38] framework to categorize UAV images of runway pavement according to different types of defects and damage severity. In this direction, the authors of [39] presented RoadCLIP, a CLIP-based model trained on a large custom-built dataset containing multiple types of cracks and damages of road pavements. Similarly, in [40], a CLIP model was used to create an image–text dataset for pavement damage detection, which was then used to fine-tune Qwen2-VL [41] to provide dense textual descriptions of pavement condition.

Despite the interesting results reported in the literature, existing approaches face limitations. Most typical image-only detection methods are restricted to identifying a specific type of defect, such as cracks. Furthermore, the evaluation procedure is usually based on simplified cases, where crack instances are captured from close range with a homogeneous background. Realistic inspection scenarios involve different types of defects, often mixed, appearing on varying backgrounds under different illumination conditions and texture variations. Conversely, VLM-based approaches operate under a more holistic paradigm, examining more complex and realistic scenarios with multiple damage types and diverse capturing conditions. Nevertheless, the provided global textual descriptions correspond to a coarse estimation of damage severity, without offering accurate localization, which limits the ability to design tailored countermeasures based on the defect type, density, geometry, and position.

In this context, our proposed framework uses well-established segmentation models to reliably detect multiple pavement surface defects at pixel level. This output supports the quantitative estimation of defect attributes such as size, shape and spatial distribution, which are fundamental to pavement condition assessment. Meanwhile, the use of data acquired by UAVs allows the framework to operate under realistic inspection conditions, accommodating the variations in texture, lighting, and defect appearance that are typically encountered in operational environments.

## 3. Materials and Methods

### 3.1. Overview

Our goal is to develop a framework for automatic inspection of runway pavement condition, with a particular focus on estimating the density and distribution of surface defects to support detailed condition evaluation. The proposed framework integrates UAV-acquired imagery, AI-based defect detection, and a GIS-driven condition assessment tool to form a complete system for automated inspection and maintenance decision support.

**Figure 1:**
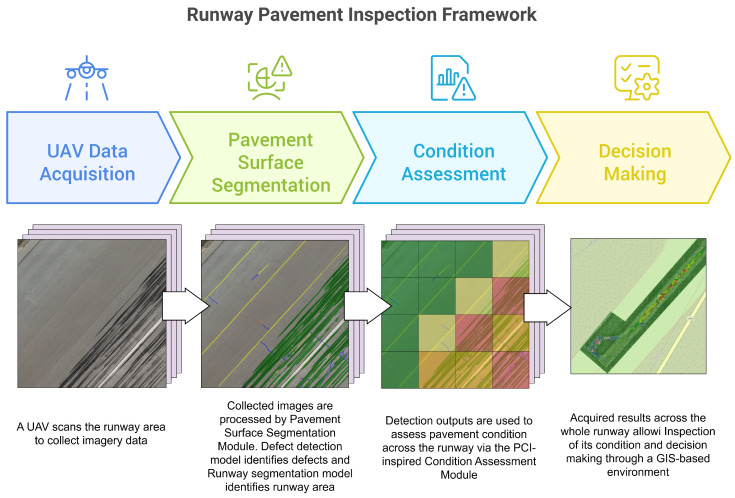
Overview of the proposed runway pavement inspection framework, illustrating the end-to-end workflow from UAV-based data acquisition to AI-driven pavement surface segmentation and GIS-based runway condition assessment.

As illustrated in Figure 1, the workflow begins with a UAV equipped with a high-resolution RGB camera that systematically surveys the runway surface to acquire visual data. The collected imagery is processed by a deep learning-based defect detection module that performs semantic segmentation to identify and localize multiple types of pavement surface defects. Semantic segmentation is employed instead of bounding-box detection to provide pixel-level information, enabling accurate estimation of defect geometry, extent, and spatial distribution.

The output of the segmentation model is subsequently integrated into a GIS-based condition assessment environment, where georeferenced defect maps are organized, visualized, and analyzed. Within this environment, defect attributes such as severity, size, and density are quantified to support pavement condition evaluation. Inspired by the principles of the ASTM Pavement Condition Index (PCI), the proposed approach derives density-based condition indicators that assist in maintenance prioritization and support condition monitoring across repeated inspections.

The following sections describe each technical component of the framework in detail.

### 3.2. Pavement Surface Segmentation Module

The pavement surface segmentation module constitutes the first processing stage of the framework, translating the raw UAV imagery into pavement surface identifications. This stage performs two complementary segmentation tasks: a. pixel-level defect segmentation and b. extraction of the runway pavement area. The former identifies and categorizes pavement defects, while the latter delineates the valid region of interest for computing accurate and meaningful defect metrics.

We use two separate segmentation models to explicitly decouple runway localization from defect identification, as the two tasks operate at different semantic and spatial scales. Runway segmentation is a coarse, high-level task that primarily relies on global appearance cues such as texture and color, whereas defect segmentation requires fine-grained analysis of localized structural patterns and defect-specific characteristics. Jointly addressing both tasks within a single model would introduce semantic ambiguity, since defects are part of the runway surface itself, and lead to a strongly imbalanced training signal due to the inherent dominance of runway pixels.

To address this issue, a dedicated model trained on detailed pixel-level annotations is used for defect segmentation, while runway areas are extracted using a robust off-the-shelf segmentation model operating in a zero-shot manner. The resulting defect and runway masks are subsequently combined to generate consistent and meaningful inputs for the runway condition assessment stage.

#### 3.2.1. Defect Detection Model

As illustrated in Figure 1, the defect detection module processes the UAV-acquired runway images and produces pixel-wise segmentation masks that categorize pavement surface anomalies. This component constitutes the core analytical stage of the framework, providing the detailed geometric information required for subsequent defect quantification and condition assessment.

The module is implemented using a U-Net-based architecture [42] for semantic segmentation, using an EfficientNet encoder. The encoder–decoder structure with skip connections enables the model to simultaneously preserve very fine crack-like patterns and capture larger-scale structures such as joints and tyre-mark streaks together with their surrounding pavement context, which is crucial for reliably detecting thin, low-contrast defects and distinguishing them from visually similar background textures. The architecture is also computationally efficient, allowing effective training on moderate-sized annotated datasets and practical deployment in the real world.

In this framework, the network assigns each pixel to one of the predefined defect classes—crack, joint (repaired crack), construction joint, tyre mark, or background—yielding a dense segmentation map suitable for subsequent spatial analysis. These segmentation outputs form the basis for computing defect attributes such as extent, density, and spatial distribution, which are later aggregated to derive the severity indicators used in the PCI-inspired condition assessment process.

Further details regarding model training, dataset usage, and optimization settings are provided in Section 4.

#### 3.2.2. Runway Segmentation Model

**Figure 2:**
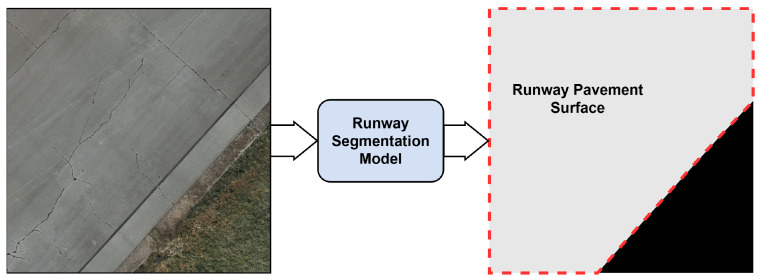
Example of runway area segmentation. A UAV-acquired image is processed by the runway segmentation model to produce a binary mask that delineates the operational runway pavement surface (gray) from non-operational areas (black).

To ensure that defect quantification and severity estimation are computed exclusively over the operational pavement surface, an additional segmentation model is used to extract the runway area from each UAV-acquired image. As illustrated in Figure 2, this model generates a binary mask that delineates the pavement region, excluding surrounding terrain and other non-operational areas.

For this purpose, we employ the Segment Anything Model 3 (SAM3) [43] in an off-the-shelf configuration without task-specific fine-tuning. SAM3 supports open-vocabulary segmentation through text-based prompting, allowing segmentation of the runway surface using simple semantic descriptions. This capability enables reliable runway extraction across varying imaging conditions without requiring explicit visual prompts (e.g., bounding boxes or point annotations) or task-specific fine-tuning. The resulting runway mask is combined with the defect segmentation output to restrict analysis to valid pavement pixels and ensure that defect density metrics and the derived PCI-inspired condition indicators are calculated solely over the inspected runway pavement surface in the downstream assessment module.

### 3.3. PCI-Inspired Condition Assessment Module

The condition assessment module integrates the outputs of the segmentation stage into a spatially organized representation of runway pavement condition. The UAV-acquired images and their geopositioning metadata are first used to construct an orthomosaic of the runway pavement surface, which is subsequently divided into sample units following the ASTM D5340 airfield pavement condition assessment methodology. Within each unit, the defect-class masks and the pavement-region segmentation mask provide georeferenced, pixel-level information on defect type, extent, and spatial distribution. This integration enables consistent visualization and interpretation of runway surface condition within a geographic context and supports the automation of established pavement evaluation workflows, forming a digital, spatially referenced representation suitable for maintenance planning.

According to the ASTM Pavement Condition Index (PCI) methodology [12], pavement evaluation requires identifying distress types, determining their extent within each sample unit before converting these into deduct values for condition scoring. A key component of this process is distress density, defined as the proportion of the pavement sample unit affected by a given defect type. In traditional PCI surveys, these quantities are derived manually through visual inspections. Our approach retains the underlying principle that distress density is a core indicator of pavement condition and automates its computation through UAV imagery and AI-based segmentation.

For each sample unit, the module computes defect densities as the ratio between the number of pixels assigned to each defect class and the total number of pixels belonging to the runway pavement surface in that unit.

This is formally defined as

(1) $$Defect\;Density=\frac{N_{\mathrm{defect}}}{N_{\mathrm{runway}}},$$

where $N_{\mathrm{defect}}$ denotes the number of pixels assigned to a given defect class and $N_{\mathrm{runway}}$ denotes the number of pixels corresponding to the runway pavement area within the sample unit. By aggregating pixel-level detections over each sample unit, the resulting defect density provides a stable area-based indicator and serves as a direct analogue to PCI’s measure, enabling precise, high-resolution quantification of surface condition. Based on these density values—and inspired by the PCI logic of associating extent and severity—the module characterizes the condition level of each sample unit, as summarized in Table 1.

This classification scheme provides a structured, PCI-inspired representation of pavement condition, assigning each sample unit to a condition class based on the computed distress density thresholds. The selected condition thresholds were defined based on recommendations from airport field experts [44] and were chosen as a practical categorization that provides sufficient discrimination for operational decision-making. The proposed framework is modular, allowing the number of condition levels and associated thresholds to be adjusted to meet specific operational or regulatory requirements. Additionally, while the current assessment is based on area-based defect density, the proposed framework is designed to preserve geometric information (e.g., length and orientation) when georeferenced imagery is available, enabling geometry-based analyses to be incorporated through post-processing if required.

The resulting severity classes are visualized as color-coded tiles over the runway pavement orthomosaic, producing a unified and interpretable condition map. Embedding this scheme within the proposed inspection framework enables clear interpretation of pavement condition, facilitates comparison across successive inspections, and supports maintenance planning by linking observed deterioration patterns with appropriate maintenance strategies.

## 4. Results

### 4.1. Case Study: Zadar Airport

To evaluate the proposed runway condition inspection framework, a case study was conducted at Zadar Airport, a medium-size international airport located 7 km east of Zadar, Croatia. The airport operates under ICAO code 4D and accommodates both civil and military traffic. It consists of two asphalt runways and associated airside infrastructure, primarily serving category C aircraft such as the Boeing 737–800, Airbus A320/A319, and Dash-8 Q400.

The pavement system at Zadar Airport is representative of many regional airports: although partial resurfacing has been performed periodically, the runways and taxiways were originally constructed several decades ago. As a result, the asphalt surfaces exhibit progressive aging, heterogeneous texture, and a variety of surface defects. Currently, runway condition monitoring is carried out through manual, non-digital inspections. These characteristics make the airport a realistic and operationally relevant environment for assessing the proposed automated runway condition monitoring framework.

### 4.2. Custom-Built Dataset

A dedicated dataset was collected from the runway pavement at Zadar Airport’s to capture a wide range of surface defect conditions. Visual data were acquired using a camera mounted on a UAV and collected across two separate missions. The first mission covered a subregion of the airport and was used primarily for data collection to support defect detection model development. The second mission, conducted several months later, covered the entire runway and was used for system-level evaluation of the proposed framework. All critical acquisition and processing parameters for both missions are summarized in Table 2.

The collected images were acquired with sufficient front and side overlap to enable photogrammetric reconstruction. The imagery from the second mission was processed using Pix4D to generate a georeferenced orthomosaic of the runway surface. From this orthomosaic, non-overlapping tiles of 5000 × 5000 pixels were extracted and used as inputs to the proposed framework, resulting in a total of 611 analysis tiles covering the entire runway. This approach ensures spatially consistent, non-overlapping coverage of the runway surface and enables uniform, sample unit-based analysis using fixed-size tiles.

Following stakeholder guidance, five annotation classes were defined: crack, joint (repaired cracks), construction joint, tyre marks, and background. Using the Make Sense [45] annotation tool, field experts selected and annotated 200 images from the first mission and 100 images from the second mission. The resulting dataset was used to train and evaluate the defect detection model: images from the first mission were used for training, while annotated images from the second mission served as the test set. Notably, the test set images were captured at a different time, originate from a different runway region, and were acquired from a different flight altitude than the training data. This configuration provides a more challenging and realistic evaluation scenario, as the two datasets differ in scale, lighting conditions, surface texture, and defect distribution.

### 4.3. Implementation of Pavement Surface Segmentation Module

For the defect detection task, we employ a U-Net [42] architecture with an EfficientNet [46] backbone, pretrained on ImageNet [47]. The model was trained for 2000 epochs with a batch size of 12. To reduce training time, the spatial resolution of the training images was halved. Data augmentation was applied during training, including random horizontal and vertical flipping (with 50% probability), rescaling, and brightness modification. In addition, random patches of 512×512 pixels in size were cropped from the input images, significantly increasing the effective size of the training dataset. Training was performed with the Adam optimizer with a learning rate equal to $10^{-3}$ and the focal loss function, which is well suited for handling class imbalance in tasks such as defect segmentation.

For runway pavement segmentation, we deploy the state-of-the-art Segment Anything Model 3 (SAM3) [43] as a pre-trained foundation model without additional fine-tuning. Text-based prompting is used to guide the segmentation process by specifying the concept phrase “road, pavement, airport runway”.

### 4.4. Main Results: Runway Digitalization

**Figure 3:**
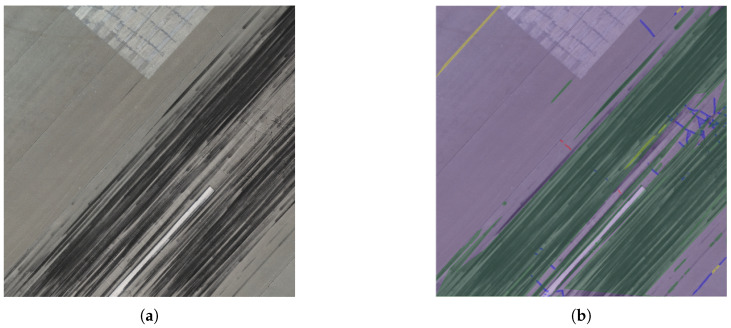
(**a**) Representative UAV image acquired during the runway inspection mission at Zadar Airport. (**b**) Corresponding segmentation output produced by the Pavement Surface Segmentation Module of the proposed framework. Red indicates cracks, blue indicates joints (repaired cracks), yellow indicates construction joints, green indicates tyre marks, and light purple indicates the non-defective pavement surface.

In this section, we present the results of deploying the proposed framework for the inspection of the entire Zadar Airport runway pavement. All 611 UAV images from the second flight mission were processed using the pavement surface segmentation module to produce the pixel-wise segmentations that highlight the detected defects and the runway surface. In Figure 3, we present a representative UAV image from the processed dataset accompanied with the outcome mask of the pavement surface segmentation module.

**Figure 4:**
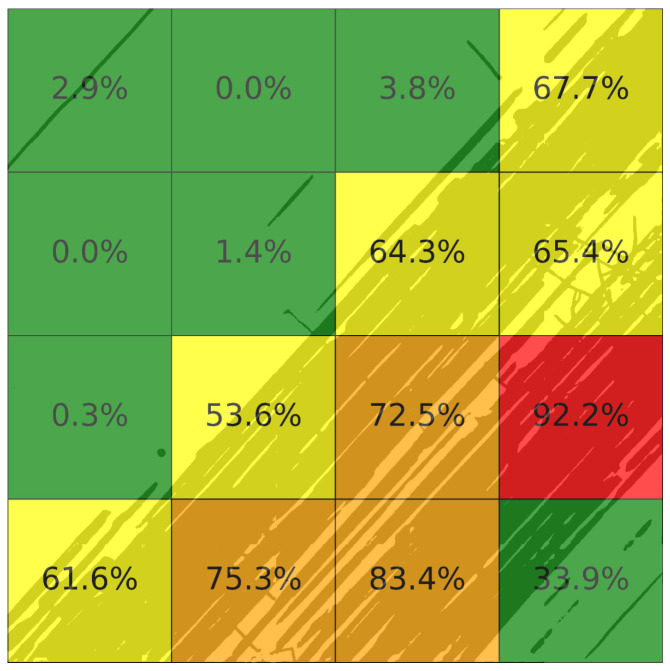
Sample-unit-level pavement condition severity categorization for the representative UAV image of Figure 3, derived from defect density values of Table 3 and classified according to the thresholds defined in Table 1.

**Figure 5:**
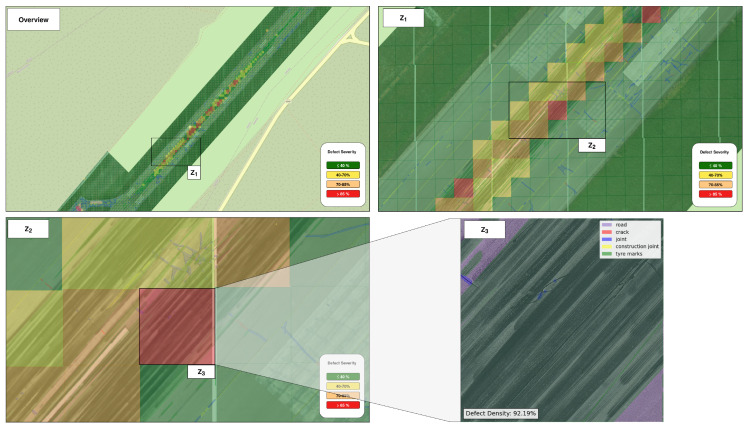
Results of the proposed framework at multiple spatial scales for the Zadar Airport runway. Starting from a runway-level overview, the GIS-based environment enables the visualization of georeferenced pavement condition information across the entire operational surface. The results clearly indicate touchdown zones which are highly affected mainly by tyre marks. Progressive zooming ($\mathrm{Overview}\to Z_{1}\to Z_{2}$) allows closer inspection of these regions, while zooming the sample-unit level ($Z_{3}$) provides detailed information on the spatial location of the detected defects and the corresponding defect density that determines each unit’s condition indicator.

These masks were then processed by the subsequent PCI-inspired condition assessment module to estimate the pavement condition at the sample-unit scale. Each image was divided using a 4×4 grid, resulting in 16 sample units per image, each covering an area of approximately 29 m^2^. This sample unit size is smaller than traditional ASTM PCI surveys [12] and allows more localized characterization of pavement condition. Table 3 summarizes the results of the defect density values for the representative UAV image in Figure 3. Similarly, Figure 4 illustrates the corresponding severity levels across each sample unit of this image, using the color-coded mapping defined in Table 1. Applying this process to all mission images yields a complete, spatially organized inspection of the entire runway pavement, as shown in Figure 5.

**Figure 6:**
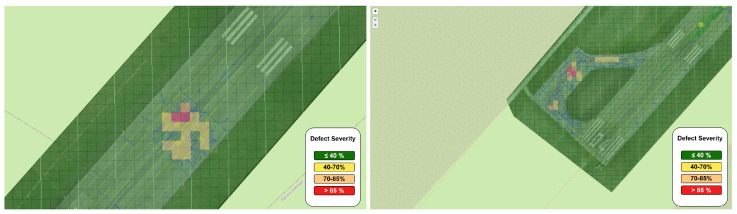
Additional close-up views of pavement condition assessment results for two runway regions exhibiting a high concentration of surface defects, predominantly joints.

Figure 5 demonstrates that the framework produces a detailed and spatially coherent representation of the pavement condition across the entire runway surface. Operating on georeferenced images enables the direct mapping of the acquired results to real-world coordinates. The combination of fine-grained defect segmentation and sample-unit condition classification enables the identification of critical pavement areas while preserving information about distress type and spatial distribution. The resulting output forms a high-resolution, georeferenced digital representation of the runway pavement condition which can serve as a digital-twin component within an airport pavement management environment, supporting routine inspection workflows and facilitating targeted maintenance planning. Figure 6 presents additional close-up views of the pavement condition assessment results for selected runway regions exhibiting an elevated number of defects.

Although the current approach relies on density-based metrics to characterize pavement condition, the use of georeferenced, pixel-level segmentation inherently links all detected surface defects to spatial coordinates. This enables straightforward extension of the framework beyond density estimation to the computation of physical attributes—such as crack length, defect area, or spatial clustering—and supports more advanced geospatial analyses within broader pavement management or digital-twin workflows.

### 4.5. Defect Detection Model Validation

In this section, we provide additional results regarding the performance of the defect detection model since it is a trainable core component of the proposed framework. In particular, we report F1 scores computed on the test set of 100 images acquired during the second UAV mission, providing a component-level assessment of the model’s segmentation capability.

The F1 score quantifies the overlap between predicted and ground-truth masks and is widely used to evaluate semantic segmentation models. In the context of runway pavement assessment, however, the role of the defect detection model is to reliably identify the presence and spatial distribution of surface defects rather than exact pixel-level correspondence. Consequently, small deviations in defect boundaries—such as thinner or partially segmented cracks—may reduce pixel-wise overlap without materially affecting the downstream severity estimation and condition assessment, as demonstrated in Section 4.4.

For evaluation, each test image was divided into non-overlapping tiles of 512×512 pixels, which were independently processed by the trained model. F1 scores were computed across the entire test dataset and are reported per defect class in Table 4.

The results indicate high performance, particularly for tyre marks and background regions. The model also demonstrates effective detection of joints (repaired cracks) and construction joints despite the visual similarity between these classes.

The comparatively lower score observed for the crack class reflects the inherent complexity of crack detection task, which is influenced by the wide variability in crack morphology, the inherent class imbalance present in the dataset, and the nature of the evaluation metric itself. The F1 score relies on strict pixel-wise agreement between predictions and ground-truth annotations and penalizes even minor boundary deviations. This mostly affects thin and elongated structures such as cracks, where even small thickness mismatches can lead to substantial score degradation. This sensitivity of pixel-based segmentation metrics for thin structures is well known and has been discussed in the literature [48,49].

In this light, we additionally evaluate the performance of the defect detection model for the crack, joint, and construction joint classes, which share to some extent similar geometric characteristics, using the Boundary F1 (BF) score [50]. BF score is a boundary-aware metric that assesses how accurately the predicted masks capture the shape and geometry of defect instances, rather than relying solely on pixel-wise overlap. The corresponding results are reported in Table 5.

**Figure A1:**
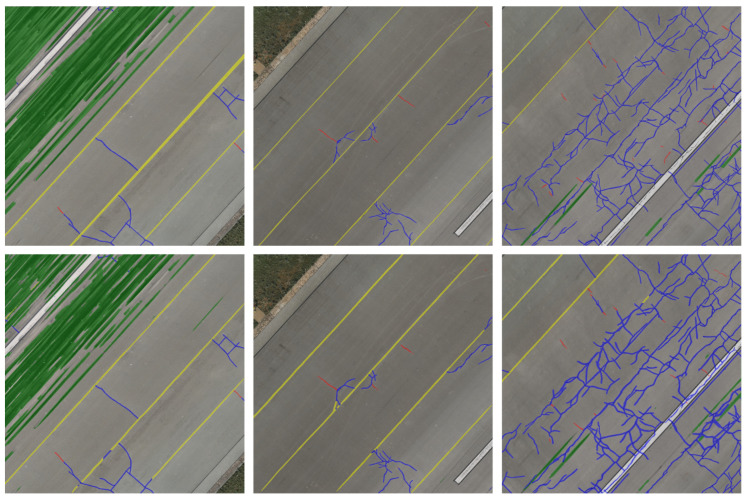
Qualitative results of the defect detection model. The first row shows ground truth-annotated masks, while the second row presents the corresponding detection outcomes. Cracks are highlighted in red, joints in blue, construction joints in yellow, and tyre marks in green.

Table 5 shows that the BF scores are significantly higher than the standard F1 scores reported in Table 4. This indicates that, although exact pixel-level alignment may not always be achieved, the proposed model accurately captures the structural layout and boundaries of critical defect instances, as also demonstrated in Section 4.4 and Figure A1 in Appendix A. Consequently, the defect detection model provides reliable localization of runway surface defects observed in the inspection flights, fulfilling its role as a core component of the proposed runway pavement condition assessment framework.

## 5. Conclusions

In this work, we presented an end-to-end framework for the automation and digitalization of airport runway pavement inspection. By integrating UAV-based data acquisition with two key components, AI-driven pavement surface segmentation and a PCI-inspired condition assessment process, the proposed system produces a detailed, georeferenced representation of runway pavement condition that serves as a practical digital-twin component within an airport pavement management environment.

A real-world case study at Zadar Airport was used to evaluate the framework. The pavement identification and defect detection modules successfully delineated the runway pavement surface and identified several categories of pavement distresses, including cracks, joints, construction joints and tyre marks, while characterizing their spatial distribution. Although performance varied across classes, particularly for fine crack-like distresses, the combined segmentation outputs provided the level of detail required to automate sample-unit condition estimation following PCI-like principles. The use of georeferenced imagery enabled the resulting condition indicators to be mapped directly to their physical locations on the runway, facilitating localized pavement assessment and supplying a structured digital layer suitable for incorporation into airport pavement management or digital-twin workflows. In addition, the pixel-level output provides a basis for computing physically meaningful distress attributes, such as crack length, crack width, or crack connectivity, which can further strengthen quantitative pavement condition analysis and align the framework more closely with established engineering assessment practices.

Overall, this work provides a solid foundation for an intelligent, automated, and data-centric approach to airport pavement condition assessment, demonstrating how UAV imaging, AI-based segmentation, and georeferenced analysis can contribute to emerging digital-twin practices in airport infrastructure management. Future work will focus on enabling predictive maintenance capabilities by incorporating historical inspection data to estimate future runway conditions and support proactive decision-making. Such a risk-based strategy is anticipated to optimize resource allocation and facilitate timely maintenance interventions, thereby enhancing the overall longevity of the runway infrastructure. In addition, foundation models will be explored in unsupervised or weakly supervised, class-agnostic settings for anomaly discovery and zero-shot label generation. Complementing anomaly detection, future research may investigate the integration of explainability mechanisms within digital-twin-enabled inspection pipelines by linking AI predictions to in-situ spatial context and maintenance decisions. Further improvements to the AI component are also anticipated, including the use of larger and more diverse training datasets, as well as the adoption of more advanced model architectures. These developments are expected to enable the system to address a wider range of inspection and maintenance tasks, including the detection of additional distress types, foreign object debris, and deformation-related anomalies, and thereby increase its utility within airport pavement management workflows.

## Figures and Tables

**Table 1:** Runway categorization based on defect density (%) values. The status categories are associated with the corresponding color scheme for visualization in the condition map.

Status	Categorization	Defect Density (%)
ine	Good	0–40
	Satisfactory	40–70
	Fair	70–85
	Poor	85–100

**Table 2:** UAV mission configuration and analysis parameters.

Category	Parameter	Value
Platform	UAV model	DJI Zenmuse Matrice 300 RTK (Shenzhen, China)
	Camera model	Zenmuse P1 (Shenzhen, China) (35 mm)
	Camera type	Full-frame RGB
Scanning Settings	Image resolution	4000×3000 pixels
	Field of view (FOV)	63.5^°^
	Image frame rate	0.7 s
	Image overlap	70% front/70% side
Flight Mission 1	Flight altitude	20 m
	Flight duration	20 min
Flight Mission 2	Flight altitude	32 m
	Flight duration	60 min
Analysis Parameters	Tile size	5000×5000 pixels
	Ground sampling distance (GSD)	∼0.00431 × 0.00431 m (∼1.86 × $10^{-5}$ m^2^/pixel)
	Area per tile	∼21.6 × 21.6 m (∼464 m^2^)
	Area per sample unit (4 × 4 grid)	∼5.4 × 5.4 m (∼29 m^2^)
	Georeferencing	EPSG: 3765

**Table 3:** Defect density values per sample unit for the representative UAV image shown in Figure 3, computed using the PCI-inspired condition assessment module.

SU	Crack Density (%)	Joint Density (%)	Construction Joint Density (%)	Tyre Mark Density (%)	Overall Defect Density (%)
ine 1	0.00	0.00	2.87	0.00	2.87
2	0.00	0.00	0.00	0.00	0.00
3	0.00	0.65	0.00	3.15	3.80
4	0.00	0.11	0.17	67.38	67.66
5	0.00	0.00	0.00	0.00	0.00
6	0.16	0.00	0.00	1.19	1.35
7	0.00	0.00	0.00	0.25	0.25
8	0.00	0.00	0.58	63.68	64.26
9	0.00	0.00	0.00	53.64	53.64
10	0.00	4.55	0.22	60.60	65.37
11	0.05	0.14	0.55	71.72	72.45
12	0.00	0.20	0.00	92.00	92.19
13	0.00	0.00	0.00	61.62	61.62
14	0.00	0.92	0.00	74.36	75.29
15	0.00	0.00	0.00	83.35	83.35
16	0.00	0.88	0.20	32.85	33.92

**Table 4:** Evaluation results of the defect detection model in terms of F1 score (%) for each class.

Classes					Mean F1
**Crack**	**Joint**	**Constr. Joint**	**Tyre Marks**	**Background**	**w/o Background**
56.99	69.73	69.70	87.29	99.09	70.92

**Table 5:** Evaluation results of the defect detection model, in terms of Boundary F1 score (%), for crack, joint and construction joint classes.

Classes			Mean
**Crack**	**Joint**	**Constr. Joint**	**Boundary F1**
73.52	83.79	87.17	81.40

## Data Availability

The data originate from internal airport operations and are therefore not publicly available. Requests for access may be directed to the authors.

## Abbreviations

The following abbreviations are used in this manuscript:

UAV	Unmanned Aerial Vehicle
GIS	Geographic Information System
PCI	Pavement Condition Index
DT	Digital Twins
AI	Artificial Intelligence
SAM	Segment Anything Model
FOD	Foreign Object Debris
ASTM	American Society for Testing and Materials

## Appendix A. Qualitative Results for Defect Detecion Model

Figure A1 presents qualitative results of the defect detection model on representative samples from the test set. The examples demonstrate the complexity of the task, where defect instances vary significantly in shape, size, and appearance. Despite these challenges, the segmentation outputs reliably detect surface defects and capture their overall geometry with sufficient fidelity for condition assessment, even when partial pixel-level deviations occur.
